# The impact of chromatin on double-strand break repair: Imaging tools and discoveries^☆^

**DOI:** 10.1016/j.dnarep.2023.103592

**Published:** 2023-11-16

**Authors:** Marit A.E. van Bueren, Aniek Janssen

**Affiliations:** Center for Molecular Medicine, University Medical Center Utrecht, Universiteitsweg 100, 3584 CG Utrecht, the Netherlands

**Keywords:** Chromatin, Fluorescence microscopy, Double-strand breaks, Nucleolus, Centromeres, Constitutive heterochromatin

## Abstract

Eukaryotic nuclei are constantly being exposed to factors that break or chemically modify the DNA. Accurate repair of this DNA damage is crucial to prevent DNA mutations and maintain optimal cell function. To overcome the detrimental effects of DNA damage, a multitude of repair pathways has evolved. These pathways need to function properly within the different chromatin domains present in the nucleus. Each of these domains exhibit distinct molecular- and bio-physical characteristics that can influence the response to DNA damage. In particular, chromatin domains highly enriched for repetitive DNA sequences, such as nucleoli, centromeres and pericentromeric heterochromatin require tailored repair mechanisms to safeguard genome stability. Work from the past decades has led to the development of innovative imaging tools as well as inducible DNA damage techniques to gain new insights into the impact of these repetitive chromatin domains on the DNA repair process. Here we summarize these tools with a particular focus on Double-Strand Break (DSB) repair, and discuss the insights gained into our understanding of the influence of chromatin domains on DSB -dynamics and -repair pathway choice.

## 1 Introduction

In order to effectively regulate DNA within the nuclear volume, eukaryotic cells organize their genetic material in the form of chromatin, which consists of an intricate network of DNA, proteins, and RNA. The nucleosome is the smallest chromatin packaging unit in the nucleus and consists of ~146 base pairs of DNA wrapped around a histone octamer made up of two copies of four different histone subunits (H2A, H2B, H3, and H4) [1,2]. Interactions between nucleosomes, as well as between histones and DNA, contribute to the organization of nucleosomes within the three-dimensional nuclear space. Additionally, histone proteins can undergo post-translational modifications that further impact chromatin packaging and facilitate the recruitment of regulatory proteins to enhance genome function [3]. Chromatin exhibits various configurations, with some regions being more compact and transcriptionally silent, such as heterochromatin domains, and others having a more accessible structure containing actively transcribed genes, often residing in euchromatin. Heterochromatin regions are associated with ‘silencing’ histone modifications, such as tri-methylation of Histone H3 Lysine 9 or Lysine 27 (H3K9me3, H3K27me3), whereas actively transcribed euchromatin regions are often characterized by ‘active’ histone modifications, such as acetylation of Histone H3 Lysine 9 or Lysine 27 (H3K9ac, H3K27ac).

The eukaryotic nucleus is continuously exposed to DNA damage, both from endogenous as well as exogenous sources. Endogenous sources often originate from regular cellular processes, such as the production of reactive oxygen species (ROS), DNA replication errors and spontaneous chemical reactions. On the other hand, exogenous sources encompass external causes such as ionizing radiation, ultraviolet (UV) radiation, and genotoxic chemicals [4]. These insults can affect the DNA in various ways, leading to for instance Double-Strand Breaks (DSBs), Single-Strand breaks (SSBs), abasic sites or DNA inter- or intra- strand crosslinks (reviewed in [5]).

One of the most damaging types of DNA damage is a DSB, where both strands of the DNA helix are severed. Proper DSB repair plays a pivotal role in maintaining genome integrity and prevents the accumulation of DNA mutations as well as major chromosomal aberrations that could lead to cellular dysfunction and disease development, such as cancer. Eukaryotic cells mainly employ two DSB repair pathways, classical Non - Homologous End Joining (c-NHEJ) and Homologous Recombination (HR) [6]. c-NHEJ achieves repair of DSBs by the direct (re)joining of the broken DNA ends by ligation. This pathway can be used throughout the interphase of the cell cycle without the use of template DNA. Although c-NHEJ is fast, genetic information is often lost at the break site due to end-processing activity at the DNA ends [7]. In comparison, HR is a homology-directed repair pathway and generally a more accurate way of repair since it uses a homologous DNA sequence as a template for repair. HR is mainly used during the S- and G2- phase of the cell cycle, after the genome is duplicated and a sister-chromatid is present, which can be used as a homologous template [6,8]. Even though HR is promoted in G2, c-NHEJ still remains the most abundantly used DSB pathway in this cell cycle phase in mammalian cells [9]. In addition to these two main pathways, there are additional DSB repair pathways present in the cell, such as Single Strand Annealing (SSA) or Alternative-End Joining (a-EJ), also termed Micro-Homology Mediated End-Joining (MMEJ). Although less abundantly active in eukaryotic nuclei, SSA and a-EJ can be utilized as alternatives to the two main DSB repair pathways in specific contexts [8,10,11].

DSB repair needs to be coordinated within a variety of chromatin environments, from actively transcribed regions to the more compact, silenced heterochromatin regions. Initial work on understanding the impact of chromatin on DNA repair had led to the model of ‘access, repair, restore’, where DNA damage causes changes in the chromatin surrounding the damaged site. These changes were hypothesized to enhance accessibility to repair proteins and thereby promote the detection and repair of damaged DNA [12]. Since the publication of this model, a plethora of chromatin changes have been identified to occur and promote repair at the damaged site (for reviews see [13–15]). In addition to the general model of ‘access, repair, restore’, it has been uncovered in recent years that not one uniform machinery is in place for the repair of different chromatin regions. For example, DSB repair in silenced heterochromatin and actively transcribed sites were found to each depend on different histone modifying enzymes [16,17]. Moreover, DSBs in actively transcribed chromatin are more prone to undergo HR, which in turn has been hypothesized to promote faithful repair of genic regions [18]. Finally, specific, directed DSB movements have been identified to facilitate repair in a variety of repetitive DNA regions, such as the ribosomal DNA of the nucleolus [19] and pericentromeric heterochromatin [20]. A picture is emerging where the distinct molecular properties of different chromatin domains have stimulated the evolution of differential repair responses to safeguard genome integrity.

This review aims to explore the crucial role of distinct chromatin domains within the nucleus and underscore the importance of understanding the repair processes that take place within these domains. In recent decades, extensive research using advanced fluorescent imaging techniques has been dedicated to studying the DNA repair response within specific chromatin contexts. We will discuss the indispensable tools required for investigating repair within diverse chromatin domains, with a particular emphasis on employing imaging methods to analyze the DSB repair response. Moreover, we will focus our attention on the DSB repair response in three specific chromatin domains that are characterized by the presence of repetitive DNA: nucleoli, pericentromeric heterochromatin and centromeres. These domains each play an essential role in the eukaryotic cell and have gained substantial interest in the DNA repair field over the past decade. Their appeal stems, in part, from their ease of visualization and the relative simplicity of inducing DNA damage within these domains due to their repetitive content. Additionally, these regions are particularly susceptible to aberrant recombination events often resulting in the formation of abnormal chromosome structures (reviewed in [21–23]), indicating the importance of understanding the repair responses in these chromatin environments. By the end of this review, we will summarize the key insights gained from imaging studies conducted on repair mechanisms within these domains, providing valuable information for further understanding the repair response in distinct chromatin contexts.

## 2 Chromatin domains within the eukaryotic nucleus

The nucleus encompasses a variety of chromatin domains that each comprise specific molecular and biophysical characteristics that influence the underlying DNA sequences, such as their three-dimensional structure or the level of transcription. Below we briefly introduce the domains we will focus on in this review; nucleoli, centromeres, and constitutive heterochromatin and discuss the importance of maintaining their underlying DNA sequences.

### 2.1 Nucleolus

The eukaryotic nucleolus is involved in the biogenesis of the cell’s ribosomes by providing ribosomal RNA (rRNA) and pre-ribosomal particles. These RNA molecules arise from active RNA-polymerase I (RNA - pol I) dependent transcription of the many ribosomal DNA (rDNA) genes that reside within the nucleolus. rDNA of eukaryotic genomes is found in numerous identical repetitive units. For example, human ribosomal DNA contains ~300 rDNA sequences, which are integrated into the short arms of five acrocentric chromosomes (chromosome 13, 14, 15, 21 and 22) and form ‘nucleolar organizer regions’ (NORs) [24]. During the interphase of the cell cycle these five NORs coalesce into one or a few membraneless nucleoli, which form through liquid-liquid phase separation of their constituents [25,26].

**Fig. 1:**
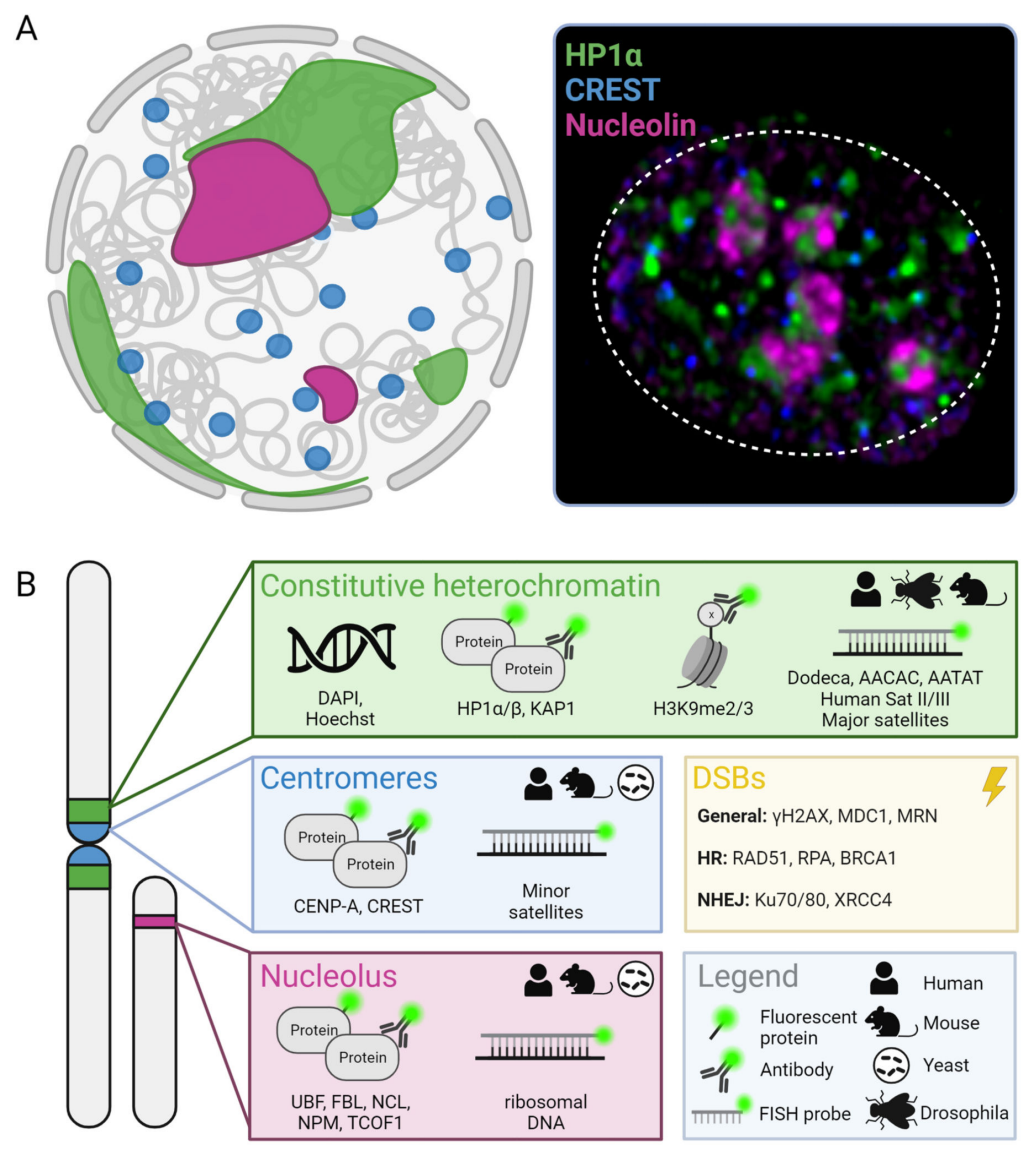
Imaging tools to analyze the DSB response in distinct chromatin domains. A) Left panel: Schematic overview of a eukaryotic nucleus illustrating different chromatin domains, with constitutive heterochromatin domains in green, the nucleolus in magenta and centromeres in blue. Right panel: Representative image of human Retinal Pigment Epithelial-1 cell nucleus immuno-stained with anti-HP1α (green), CREST (blue) and anti-nucleolin (magenta) to visualize constitutive heterochromatin, centromeres, and the nucleoli respectively. B) Each box gives an overview of imaging approaches to visualize the different chromatin domains during DSB repair; constitutive heterochromatin (green), centromeres (blue) and nucleoli (magenta). DAPI and Hoechst can be used in mouse and Drosophila cells to visualize the constitutive heterochromatin chromocenters. Different FISH probes have been used to visualize repeats in constitutive heterochromatin in a variety of model organisms; AACAC and AATAT probes in Drosophila, Human Satellite III (Human Sat.III) probes in human cells, and major satellite repeats probes in mouse. Minor satellite repeats are enriched in mouse centromeres. A selection of proteins which can be used to visualize DSBs are shown (yellow box). HR = Homologous Recombination, NHE*J* = Non-Homologous End Joining.

In mammals and other higher eukaryotes, the nucleolus contains three functionally and structurally distinct regions, termed fibrillar center (FC), dense fibrillar component (DFC) and granular component (GC) [27]. Transcription of rRNA concentrates itself within the FC and FC-DFC border. Subsequently, rRNA processing occurs in the DFC, whereas the first steps of ribosome assembly occur within the GC. This compartmentalization of rDNA transcription and downstream processing results in the containment of active rDNA regions within the FC or FC-DFC border, associated with more active histone marks as well as low levels of DNA methylation. Silenced rDNA genes on the other hand contain silencing histone modifications and can be found in the periphery of the nucleolus, which closely associates with heterochromatic sequences [28] (Fig.1).

Understanding how DNA damage in the nucleolus is being repaired has intrigued many researchers over the past decades. Maintaining an intact nucleolar and rDNA structure is essential for organismal health and preventing disease onset [29–31]. rDNA is often exposed to DNA damage, which is thought to be due to its high transcriptional activity and repetitive content, thereby creating situations of replication stress [23]. In line with this, rDNA is a frequently rearranged chromosomal region within solid tumors [32]. It is therefore imperative to understand how these nucleolar structures are being maintained in the presence of DNA damage and what the role of nucleolar components is in the repair response. With its specific compartmentalization, differential transcriptional activities and abundant repetitive sequences, the nucleolus requires highly specific repair mechanisms.

### 2.2 Centromeres

Centromeres are chromosomal structures essential for proper chromosome segregation upon cell division in eukaryotes. Centromeric sequences can incorporate the histone H3 variant Centromere Protein – A (CENP-A), which epigenetically marks the centromere [33]. Centromeric chromatin in several primates and mouse species also includes Centromere Protein – B (CENP-B), which recognizes a specific 17 base pair sequence found in centromeric DNA [34,35]. These epigenetically defined centromeres promote the recruitment of additional centromere proteins that together form a platform termed the Constitutive Centromere-Associated Network (CCAN) [33]. The centromere is required for assembling the kinetochore in mitosis, a proteinaceous structure needed for the binding of microtubules emanating from the spindle poles. These kinetochore - microtubule interactions are essential for proper orientation of the sister chromatids during mitosis, and subsequent correct chromosome segregation upon cell division. Loss of centromere function results in mitotic errors and the appearance of abnormal chromosome numbers in daughter cells, termed aneuploidy.

Centromeres often contain repetitive sequences, such as alpha-satellite repeats in human cells or minor satellite repeats in mouse cells [36]. The presence of many repetitive sequences makes centromeres susceptible to aberrant replication and subsequent defects in recombinational repair, indicating the importance of properly maintaining this domain [22,37]. Moreover, improper centromere repair can directly result in the formation of abnormal chromosome structures, such as translocations or dicentric- and acentric- chromosomes, associated with disease development [37]. It is therefore essential that this structure remains intact and that we understand the mechanisms that safeguard centromeric DNA structure in the presence of DNA damage.

### 2.3 Constitutive heterochromatin

Constitutive heterochromatin can be mainly found around the centromeres as well as the regions surrounding the telomeres, called pericentromeres and subtelomeres respectively. Constitutive heterochromatin is abundant in eukaryotic genomes, where it covers ~25–90 % of the genome [38–40]. Molecularly, constitutive heterochromatin consists of high levels of H3K9me2/3, which is bound by Heterochromatin Protein 1 (HP1 α in mammals, HP1a in Drosophila melanogaster). HP1α and its interacting proteins promote the clustering and condensate formation of heterochromatic sequences [41,42], thereby creating a dynamic, but stable compartment that enhances silencing of its underlying repetitive sequences [43]. Depending on the organism or tissue type, heterochromatin often forms one or a few membraneless domains within the nucleus [44].

The constitutive heterochromatin compartment is essential for the maintenance of genome stability by supporting centromere- and telomere- structure, as well as in preventing deleterious expression of repetitive sequences [21]. Heterochromatic repetitive sequences are mostly composed of tandem satellite repeats and transposons. Disrupting heterochromatic satellite silencing results in the formation of RNA-DNA hybrids that can subsequently cause genome instability [45, 46]. In line with these important functions for heterochromatin in maintaining genome stability, loss of heterochromatin integrity promotes tumor formation in mice [47] and is associated with aging and cancer in humans [48,49]. Moreover, H3K9me3-enriched regions are often associated with a high mutational load in cancer, indicating their susceptibility to aberrant DNA repair [50,51]. Indeed, the presence of many homologous repetitive sequences present on non-homologous chromosomes makes heterochromatin susceptible to aberrant recombinational repair in the presence of DSBs [20,52], indicating the importance of properly orchestrating repair within this compact domain.

In conclusion, the eukaryotic nucleus consists of various chromatin domains, each playing a crucial role in cellular functioning. Exploring how these chromatin regions tackle DNA damage has been a fascinating area of research, leading to valuable discoveries in recent years. In the following section, we delve into the prerequisites for conducting fluorescent imaging of the repair response within specific chromatin environments, again emphasizing on studying DSB repair in the repetitive regions of eukaryotic genomes found in nucleoli, centromeres, and constitutive heterochromatin.

## 3 Imaging the DSB response in distinct chromatin domains

### 3.1 Choice of model system

There are abundant options for selecting a model organism to perform imaging of DNA repair within a particular chromatin domain (Fig.1). Historically, the study of chromatin changes at DNA damage sites has heavily relied on the use of the budding yeast *Saccharomyces cerevisiae* [53]. Indeed, significant insights into specific DSB movements within rDNA sequences were initially obtained from budding yeast [19]. Although budding yeast has greatly contributed to our understanding of chromatin dynamics at DSB sites, its chromatin domain composition is less complex than many other model systems. When aiming to comprehend DNA repair in the context of additional chromatin domains such as constitutive heterochromatin or facultative heterochromatin, other model organisms, such as the fruit fly *Drosophila melanogaster* or mouse cells could be considered [54,55]. Both Drosophila and mouse cells feature well-defined heterochromatin domains that prominently appear as cytological structures in interphase cells. When examining repair processes within the inactive X chromosome, female mammalian cells provide a suitable experimental setup [56], while imaging of repair in the context of nucleoli is commonly conducted in human cells due to their highly visible nucleolar structures [57]. For studying repair within lamina-associated heterochromatin domains, model systems such as C. elegans or various human cell lines could be preferred since these exhibit notable localization of heterochromatin at the nuclear periphery [58, 59]. In conclusion, the selection of model system depends on the specific chromatin domain of interest (Fig. 1). Nonetheless, when aiming to establish a fundamental understanding of DNA repair within chromatin, it can be advantageous to employ a diploid system without apparent chromatin alterations to ensure the investigation of these molecular repair mechanisms in a physiologically relevant context.

### 3.2 Visualizing DSB repair in specific chromatin domains

Imaging the DNA damage response in specific chromatin domains requires the use of specific fluorescently labeled chromatin- and repair-markers. We have generated an overview of fluorescent tools that have been used to visualize DSB repair in different chromatin domains in a variety of studies and model organisms in both fixed and live imaging analyses (Fig. 1).

Each chromatin domain is characterized by specific histone modifications, DNA sequences or chromatin proteins, which can be employed to specifically label the respective domain. Nucleoli can for example be marked using immunofluorescence staining or live fluorescent tagging of the nucleolar-specific proteins Upstream Binding Factor (UBF), Fibrillarin or Treacle [60–62]. For imaging DNA damage repair at centromeres, fluorescent imaging of centromeric proteins, such as CENP-A or CENP-B is the most conventional choice [63]. However, the histone subunit CENP-A is only integrated in specific satellite repeat regions, which could lead to an incomplete visualization of the centromere [64]. Moreover, it has been found that CENP-A recruitment is intimately linked to the repair process, also at non-centromeric break sites [63,65, 66]. To overcome these potential problems with identifying centromeres based on CENP-A visualization, the mammalian centromere-specific CREST antibody could be employed as well, which provides a simple centromere-visualization tool in fixed cells [63,67,68]. Alternatively, fluorescent labeling of CENP-B can be used in live mammalian cells to visualize the centromere.

For performing live imaging of repair in constitutive heterochromatin the most convenient choice is the use of fluorescently tagged HP1 (HP1a in Drosophila, or HP1α in mammalian cells), since this protein brightly marks heterochromatin domains within the nucleus [20,69]. Hoechst or DAPI staining can also be considered for visualizing AT-rich heterochromatic sequences in live or fixed Drosophila and mouse cells. Moreover, immunofluorescence staining for H3K9me2/3 allows the visualization of pericentromeric sequences in many eukaryotes and can effectively distinguish constitutive heterochromatin from the rest of the chromatin [9,20,56,70–81].

The highly repetitive nature of rDNA and (peri-)centromeric sequences also allows for the easy use of DNA - Fluorescence In Situ Hybridization (FISH) probes to visualize these sequences. When combined with immunofluorescence for specific DNA repair proteins, these FISH probes serve as a very specific way to visualize the damaged repetitive DNA [16,20,61,73,75,77,82].

To visualize localization of break sites to specific nuclear structures, such as the nuclear lamina or nuclear pores, researchers have also employed live- or fixed- imaging of specific lamina – or nuclear pore proteins-, such as Lamin B in human cells [58] or Nup153 in Drosophila cells [75].

Finally, the choice of DNA repair marker depends on the type of DNA damage and repair pathway one is investigating (for overview, see [83] and Fig. 1). The phosphorylation of histone H2AX (γH2AX in mammals, γH2Av in drosophila) is a standard DNA damage marker used for fixed immunostainings. This is primarily because it rapidly emerges following DNA damage and spreads across megabases in both directions from the break site, rendering the signal easily detectable [84–88]. However, caution should be used when solely using phosphorylation of H2AX as a marker for DNA damage. Absence of yH2AX signal does not necessarily reflect the absence of DNA damage, since its phosphorylation requires active PI3 kinase signaling [86]. Therefore, defects in for example Ataxia Telangiectasia Mutated (ATM) or Ataxia Telangiectasia And Rad3-Related Protein (ATR) could abolish yH2AX appearance in the presence of DNA damage. Moreover, yH2AX signaling has also been suggested to occur in the absence of DNA damage [87,89]. As an alternative, proteins more downstream in the DNA Damage Repair (DDR) signaling cascade can be used to visualize the repair process, such as players specific for either Homologous Recombination (HR) or Non-Homologous End Joining (NHEJ) (Fig.1).

The emergence of CRISPR-Cas9 genome editing has introduced a highly advanced method for visualizing repair and chromatin responses by enabling the integration of fluorescent tags at the endogenous gene loci of any repair- or chromatin- related protein of interest. However, researchers still extensively employ transient expression of fluorescent proteins and immunofluorescence techniques to study repair proteins, which consistently contribute valuable findings in the field.

Nucleoli, centromeres, and constitutive heterochromatin are prominent structures within the nucleus that are readily observable, making them convenient targets for studying the DNA repair response using fluorescent imaging techniques. However, when imaging the repair response in these domains using fixed specimens, it is important to consider that DNA repair is a highly dynamic process. For example, DSBs in constitutive heterochromatin and nucleoli have been identified to undergo specific spatio-temporal dynamics, where break sites move outside of the respective chromatin domain to finalize repair [19,20,61]. When conducting repair analysis on fixed cells, it is crucial to consider the potential oversight of certain repair events due to the rapid movement of DSBs outside their respective domains.

## 4 Tools to induce DNA damage in different chromatin domains

**Fig. 2:**
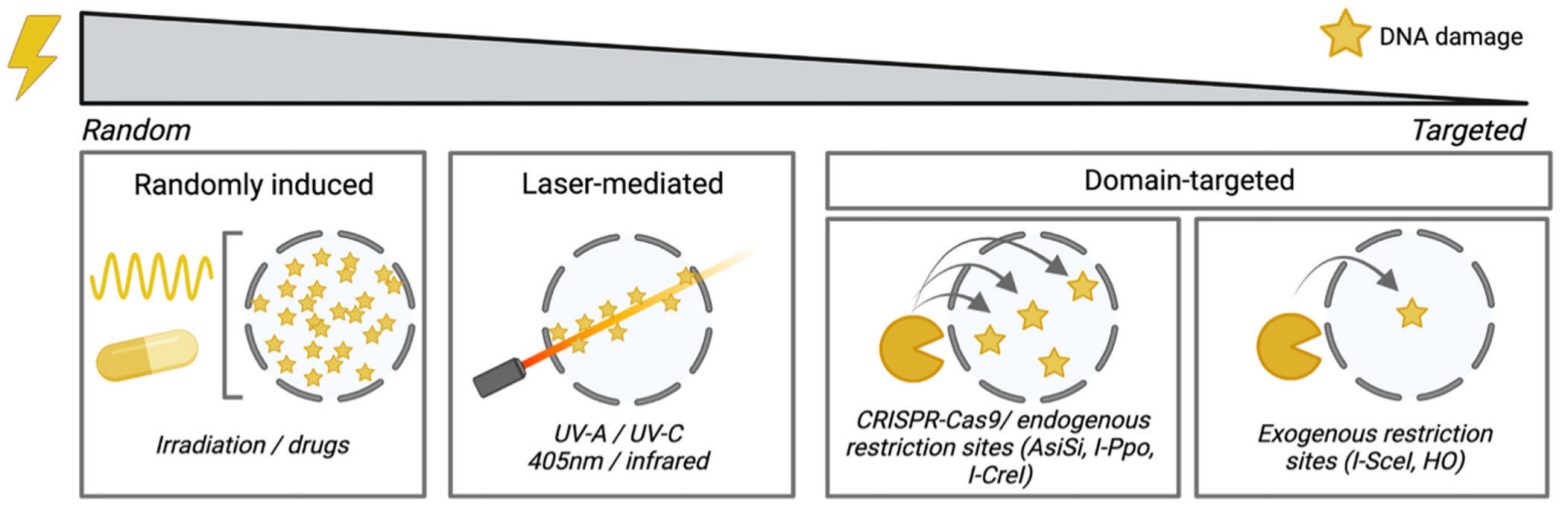
Tools to induce DNA damage in different chromatin domains. An overview of the different techniques used to induce DSBs in eukaryotic cells. DSB methods range from random induction (left) to more targeted approaches (right). DNA damage is depicted by the yellow stars.

The investigation of DNA repair begins with selecting an appropriate method for inducing damage. The methods employed to induce DNA damage range from randomly inducing multiple breaks to a more focused approach of inducing a specific number of DSBs at pre-determined DNA locations (also reviewed in [90,91]). In this section, we provide a summary of various approaches that have been developed to induce DNA damage either throughout the entire genome or within specific domains (Fig.2). Each of these approaches possesses its own advantages and disadvantages, which will be discussed.

### 4.1 Randomly - induced DNA damage

Global induction of DNA damage refers to the induction of DNA lesions throughout the genome, often using genotoxic agents such as irradiation or mutagenic drugs. Many of these reagents or irradiation techniques come with the advantage that they can be precisely timed and therefore allow analysis of the repair response within short time-intervals following damage induction.

Ionizing radiation (IR) refers to high-energy radiation that has sufficient energy to disrupt the binding of electrons to atoms, resulting in the formation of charged particles called ions. There are multiple types of IR; alpha-, beta-, X-ray-, gamma- and neutron- radiation. Exposing cells to IR leads to the formation of a variety of DNA lesions, such as DSBs, Single - Strand Breaks (SSBs) and abasic sites [92,93]. IR is often used in the field of DNA repair since it allows the rapid induction of damage. Moreover, the dosage of IR can be controlled, either through the intensity of radiation or the exposure time.

In addition to IR, DNA damage can also be induced by treatment with specific compounds that have been developed for potential use as anti-cancer chemotherapeutic drugs, such as topo-isomerase I/II inhibitors (etoposide, Adriamycin), intra- and inter-strand crosslinkers (cisplatin, carboplatin) or reagents that induce replication stress (hydroxyurea, aphidicolin) [90].

These compounds have as an advantage their relatively low costs and their usually rapid induction of DNA damage, allowing the analysis of the repair response in a timely fashion. However, the disadvantage of both IR and damaging-inducing compounds is that the damage is often induced randomly. When studying chromatin context - dependent repair mechanisms using these approaches, it is therefore important to always include a fluorescent marker that is specific for your chromatin domain of interest (Fig. 1). Additionally, certain chemicals and radiation treatments can exhibit variations in DSB induction depending on the chromatin environment. For instance, etoposide, which inhibits Topoisomerase-II, specifically enhances the occurrence of DSBs at chromosome - loop anchor regions, as well as regions with high transcriptional activity [94,95]. Similarly, IR can produce diverse patterns of DNA breakage based on the structure of the chromatin [96,97]. These differences in the efficiency of inducing DNA damage should be considered when utilizing these approaches to investigate DNA repair within different chromatin contexts.

### 4.2 Laser-mediated DNA damage induction

To achieve the same inducibility as the use of chemicals or IR, but overcome their random damage induction, various labs have made use of laser-mediated (micro) irradiation, where one is able to target damage to specific nuclear regions and follow the DNA damage response at high time-resolution. Lasers can be angled in such a way that they induce DNA damage in a small (circular, linear) region inside the nucleus [98]. Moreover, employing lasers with different wavelengths allows the induction of different types of DNA damages at any region of interest. For example, exposing cells to a UV-C (266 nanometer (nm)) laser can directly induce pyrimidine - (thymine, cytosine) dimers that are detected and repaired by the nucleotide-excision repair (NER) machinery [98]. To timely and locally induce DSBs using laser-micro irradiation, UV-A, 405 nm, infrared or heavy-ion lasers can be employed. UV-A, 405 nm and infrared exposures are often combined with a pre-sensitization step (BrdU incorporation, Hoechst treatment) to enhance the efficacy of DSB induction [98,99]. Combining the use of these lasers with imaging of specific fluorescent chromatin markers allows the direct targeting of DNA damage to any chromatin domain of interest. Indeed, visualizing constitutive heterochromatin using the DNA-dye Hoechst or expression of fluorescently tagged HP1α/β has been used in combination with laser-irradiation, thereby allowing the live analysis of the impact of DNA damage on heterochromatin composition [56,74,80,81].

Although this laser-induced DNA damage is amenable for studying DNA damage dynamics in an inducible and region-specific fashion, these treatments do induce relatively high local levels of DNA damage, which might not be entirely physiologically relevant. In addition, 405 nm, UV-A or infrared lasers induce a variety of damages (SSBs, DSBs, photolesions), which can lead to difficulties when attempting to precisely link the repair response to a specific type of damage [98].

### 4.3 Domain-targeted DNA damage methods

To be able to study the impact of pre-existing chromatin environments on DNA repair, it is often advantageous to employ approaches that induce DNA damage specifically in the chromatin domain of interest. Below we will discuss these domain-specific DSB-inducing agents that have been employed for nucleoli, constitutive heterochromatin and centromeric chromatin. Using these DNA damage methods (Fig.2) in combination with live- or immunofluorescence-based imaging of repair- and chromatin- proteins (Fig.1) has allowed for a more in-depth analysis of the impact of chromatin on DNA repair.

#### 4.3.1 CRISPR-Cas9 to induce locus-specific DSBs

The development of CRISPR-Cas9 to introduce DSBs and repair at any genomic region of interest in the eukaryotic genome has allowed scientists to explore the repair response in many different chromatin contexts [100]. In particular, the repetitive regions of rDNA and (peri-) centromeres provide a relatively straightforward target to induce and visualize DSBs using CRISPR-Cas9. For example, mouse pericentromeric heterochromatin is rich in tandem major - satellite repeats, while mouse centromeric DNA is enriched for minor - satellite repeats (Fig. 1). Designing sgRNAs that recognize major- or minor- satellite repeats leads to the formation of multiple DSBs in either constitutive heterochromatin or centromeres respectively when co-expressing CRISPR-Cas9 [67,78, 101]. Similarly, Drosophila pericentromeric heterochromatin contains specific tandem repetitive sequences, such as the Dodeca-satellite repeat region on chromosome III [102]. Expression of sgRNAs targeting the Dodeca repeat in combination with an inducible Cas9 variant results in the timed induction of DSBs specifically in pericentromeric heterochromatin in Drosophila cells [16]. Likewise, human pericentromeric heterochromatin contains Satellite II and Satellite III repeat integrations. Introducing sgRNAs specific for these repetitive sequences in human cells has allowed the targeted induction of DSBs in heterochromatin upon Cas9 expression [101].

Similar to the (peri-) centromeric regions of the genome, the repeat-rich rDNA can also be targeted by CRISPR-Cas9 to induce DSBs [60,61, 103,104]. Each of the ~300 human rDNA repeats measures around 43 kilobases in length and consists of an rRNA gene, flanked by promoter and terminator sequences, as well as an intergenic space. This mapped rDNA sequence allows for the design of rDNA-targeting sgRNAs, which can, when combined with Cas9 expression, simultaneously induce hundreds of DSBs. Conveniently, these DSBs can be easily visualized using a variety of DSB markers using either fixed or live cells (Fig. 1). Moreover, when combined with nucleolar markers, such as UBF or nucleolin (NCL), this break induction allows for the specific image analysis of DSB repair within the nucleolus [60,61].

An advantage of using CRISPR-Cas9 to induce damage in repeat-rich regions is that the visible DSBs will likely be specific for the respective chromatin domain containing the repetitive sequences. One can therefore be relatively sure that they are studying domain- specific DSB mechanisms. In addition, due to their large number, these Cas9-induced DSBs in repetitive sequences are easy to visualize. Another advantage of targeting repetitive sequences using CRISPR-Cas9 is that one can simultaneously visualize the specific chromatin domain using FISH labeling of the respective repeats [16,61,63,101,103].

However, a disadvantage of using CRISPR-Cas9 to induce DSBs in repetitive rDNA or (peri-) centromeric sequences is that many DSBs are being induced at the same time, which might not mimic a completely physiological situation. Moreover, differences in accessibility of the Cas9 enzyme in heterochromatin have been described, which could affect DSB repair kinetics [105]. In addition, CRISPR-Cas9 repair often leads to relatively slow and inaccurate repair when compared to other types of DSBs, indicating it might not completely reflect the repair process of endogenous DSBs [106].

Although the CRISPR-Cas9 system has mainly been used to induce DNA double-strand breaks, this system could in theory be adapted to induce different types of DNA damage by for example coupling a catalytically - dead Cas9 (dCas9) variant to ROS-generating proteins [107] or employing a D10A point mutation in Cas9 to generate a nickase variant [108] that could induce SSBs at repetitive sequences.

#### 4.3.2 Endogenous restriction sites

In addition to the use of CRISPR-Cas9, several exogenous restriction enzymes have been employed to induce DSBs in specific genomic regions. Three examples are the enzymes I-PpoI, AsiSI and I-CreI. I-Ppo was originally cloned from myxomycete Physarum polycephalum [109] and recognizes a specific 15 base pair sequence in the 28 S rDNA coding sequence [110] in addition to ~30 sites elsewhere in the human genome. AsiSI was originally cloned from Arthrobacter species and recognizes specific 8 base pair sequences in the genome. AsiSI has a recognition site in the rDNA locus in the 5’ external transcribed spacer of the rDNA gene [111] and additionally has ~100–200 accessible sites in the human genome [112–114]. Both I-Ppo and AsiSI-systems have been developed to allow the inducible appearance of DSBs [60,113,115], thereby facilitating the timed induction of DSBs in rDNA, which helps in the subsequent analysis of rDNA – specific DSB repair mechanisms. Finally, I-CreI, originally cloned from the green alga Chlamydomonas reinhardtii, has 24 base pair - endogenous recognition sites in both the human [110] and Drosophila melanogaster [116] 28S rDNA sequences, and can also be employed to analyze the behavior of rDNA - induced DSBs.

Similar to the use of CRISPR-Cas9 to induce DSBs in repetitive regions, all these enzymes will generate many DSBs in the nucleolus, possibly concealing a more physiological DSB response. Moreover, it should be considered that when these DSBs are perfectly repaired, the restriction site remains intact and can lead to repeated rounds of cleavage when the enzyme remains active.

However, the formation of multiple DSBs comes with the advantage of allowing the direct visualization of DSBs in rDNA for image analysis. In addition, since their target sites have been specifically mapped, one can perform ChIP-sequencing analyses to assess chromatin changes associated with these DSBs [111].

Finally, AsiSI efficiently induces DSBs in open ‘active’ chromatin regions and to a lesser extent in ‘silent’ heterochromatic regions [112–114]. With the precise mapping of accessible AsiSI target sites in the human genome, it is in theory possible to utilize AsiSI expression for live - imaging analysis of the repair response in a variety of active chromatin regions, contingent upon the use of specific fluorescent chromatin proteins. Up to now, the exploration of the repair response in other chromatin domains targeted by AsiSI has primarily focused on the identification of histone modifications, three-dimensional DNA interaction patterns as well as repair - pathway analyses [18,112,117]. However, there is potential for expanding these investigations to incorporate fluorescent live imaging in a variety of chromatin domains in the future. This expansion would allow for a more dynamic understanding of the repair processes occurring within different AsiSI-targeted chromatin domains.

#### 4.3.3 Exogenous restriction sites to induce single DSBs

To overcome the induction of many DSBs simultaneously, researchers have also employed the integration of an exogenous restriction site as a way to induce single DSBs in specific chromatin domains. The I-SceI endonuclease from *Saccharomyces cerevisiae* recognizes and cuts a unique 18 base pair sequence [118]. Combining the targeted genomic integration of an I-SceI restriction site with (inducible) expression of the I-SceI enzyme has opened up many avenues in the field of DSB repair [19,69,118–120]. For example, simultaneously introducing Tet Operon (TetO) repeat sequences next to an I-SceI cut site has allowed the visualization of specific DNA loci before and after DSB induction by co-expressing fluorescently tagged TetR, a TetO binding protein, or by performing DNA FISH on TetO repeats [19,69].

Moreover, integrating an I-SceI dependent DSB - repair reporter in either eu- or heterochromatic sites in the Drosophila genome has allowed for the in-depth image analysis of DSB movements, as well as the identification of chromatin changes and repair - pathway usage in live Drosophila tissues [16,69]. Furthermore, I-SceI cut sites have been introduced nearby budding yeast centromeric- and telomeric- sequences, as well as in human lamina-associated domains to identify movements and repair - pathway choice of DSBs in different nuclear locations within live cells [58,121,122]. The utilization of single DSB assays enables a highly focused examination of repair responses, effectively resolving the issue of a large number of DSBs arising from targeting repetitive sequences with CRISPR-Cas9, I-Ppo, I-Cre, or AsiSI. However, the visualization of individual DSB sites necessitates the use of more advanced imaging techniques with greater resolution.

## 5 Chromatin domains influence DSB dynamics

Combining inducible DNA damage tools with image analysis of repair- and chromatin- proteins (Figs.1, 2) has yielded significant insights into the impact of different chromatin domains on DNA repair processes. Below we outline several important biological discoveries that have been realized through the utilization of these tools.

### 5.1 Nucleolar reorganization at damaged sites

**Fig. 3:**
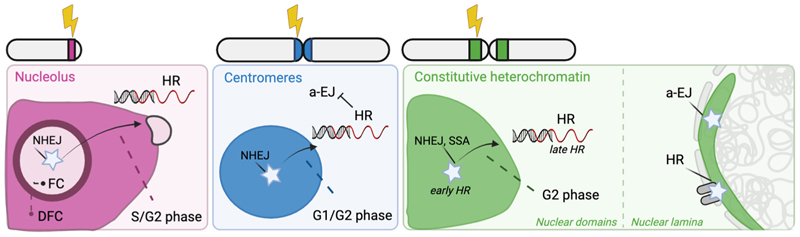
DSB dynamics in different chromatin domains. DSBs can move outside of nucleoli (magenta, left), centromeres (blue, middle) and constitutive heterochromatin (green, right) to be repaired. Specific cell-cycle phases can influence DSB movements, with nucleolar- and heterochromatic- DSB movements specifically in S/G2 and centromeric- DSB movement in both G1 and G2. Breaks in nuclear periphery- associated heterochromatin can be repaired by a-EJ (lamina) or HR (nuclear pores, dark grey). DFC = Dense Fibrillar Component, FC = Fibrillar Centre, NHE*J* = Non-Homologous End Joining, HR = Homologous Recombination, a-E*J* = alternative End Joining, SSA = single strand annealing.

**Fig. 4:**
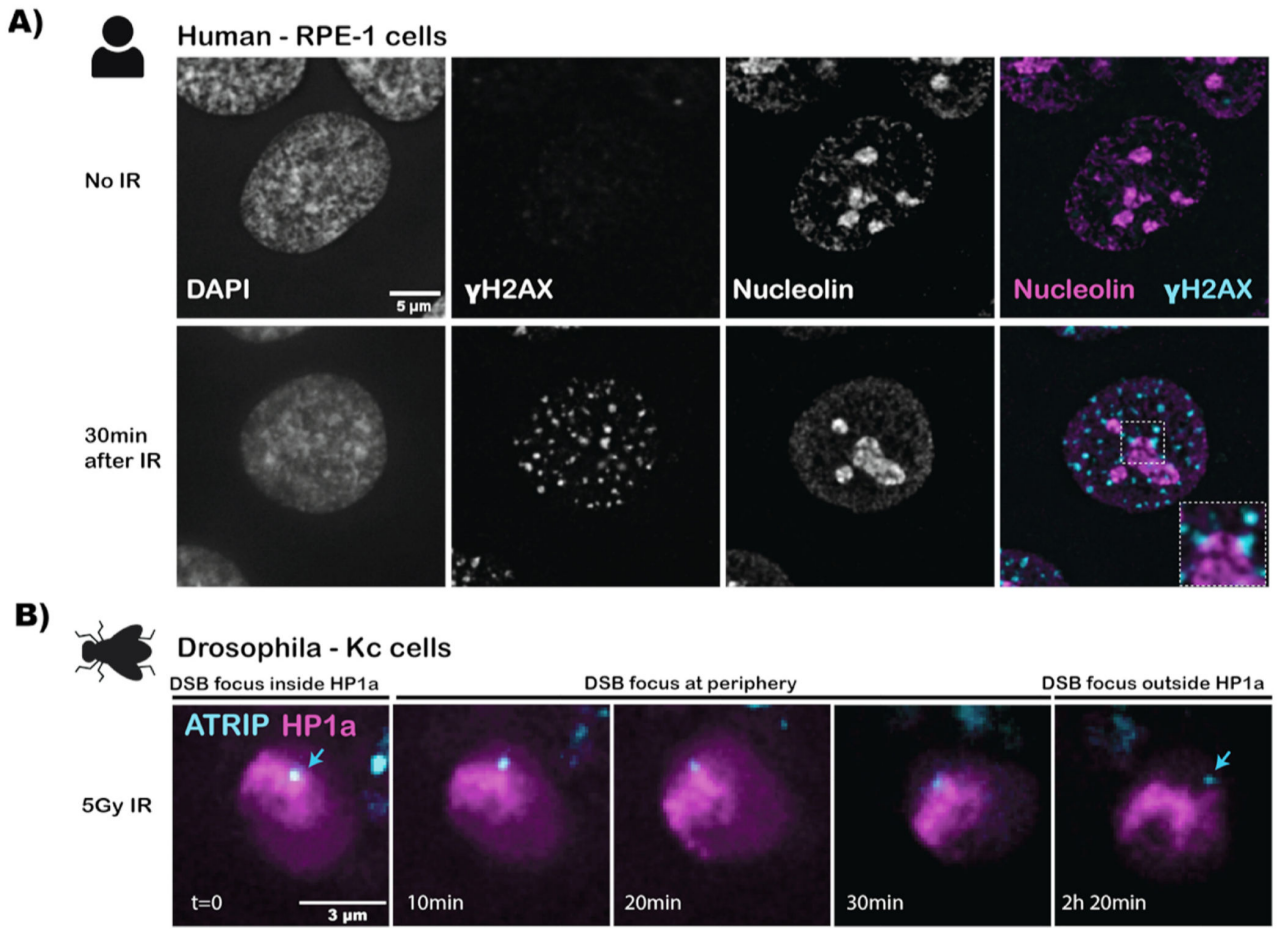
Visualizing DSB movement to the nucleolar and heterochromatin periphery. A) Immuno-fluorescence images of human Retinal Pigment Epithelial (RPE-1) cells stained with anti-γH2AX (cyan, DSBs) and anti-nucleolin (magenta, nucleolus). Top: non-irradiated cells, bottom: the presence of yH2AX foci at the nucleolar periphery 30 min after 3 Gy γ-radiation. B) Live-imaging of γ-irradiated (5 Gy) *Drosophila melanogaster* Kc cells to visualize movement of DSBs (YFP-ATRIP, HR protein, cyan) to the periphery of constitutive heterochromatin (Cerulean-HP1a, magenta).

One of the most striking findings obtained by live imaging studies in the past decades has been the identification of specific DSB movements in a variety of chromatin domains, often coinciding with specific chromatin reorganizations (Fig. 3). For example, DSBs in nucleoli undergo specific movements to the nucleolar periphery, which are thought to be necessary for proper DSB repair (reviewed in [57]). Nucleoli rearrange their composition in the presence of DNA damage (UV-lesions, DSBs), a restructuring dependent on the DNA - damage checkpoint kinases ATM and ATR [61,123,124]. These nucleolar reorganizations are characterized by the formation of nucleolar caps that form at the periphery of the nucleolar domain. These nucleolar caps contain FC and DFC regions, which are normally present in the nucleolar interior as centers for active rRNA transcription. DSBs in rDNA localize to these nucleolar caps, which are enriched for a variety of DSB proteins and are thereby thought to promote efficient rDNA repair [61,123] (Fig. 4A).

Interestingly, initial work suggested that the formation of nucleolar caps is solely dependent on the inhibition of RNA pol I transcription by DNA damage checkpoint kinases [61,123,125]. However, recent evidence revealed that DSB-associated RNA pol I inhibition does not always result in nucleolar cap formation and that in fact cap formation is specific for the S and G2 phases of the cell cycle [111]. In line with this, another study revealed that nucleolar restructuring upon DSB induction was significantly enhanced upon inhibition of repair by NHEJ, indicating that fast DSB repair by NHEJ in G1 may not require nucleolar restructuring [123], while HR in S/G2 might depend more heavily on this process (Fig. 3). Indeed, additional studies identified that nucleolar caps are enriched for HR repair proteins [60,61,103,124]. Together, these results indicate that the choice of repair pathway, as well as cell cycle - dependent factors could be contributing to specific nucleolar restructuring in the presence of DNA damage.

DSB-induced nucleolar restructuring in human cells has also been identified to depend on actin activities and the nuclear-envelope embedded LINC complex [111], indicating that actin-driven movement to the nuclear periphery is a pre-requisite for proper rDNA repair. This is in line with earlier work in yeast which revealed the movement of nucleolar DSBs to the periphery of the nucleus as well [19]. The exact role of nucleolar restructuring and peripheral movement in the repair process, whether it solely enhances HR repair efficiency, or possibly also prevents aberrant recombination between rDNA repeats by spatially separating them from each other, remains to be resolved.

Interestingly, several chromatin processes are involved in the movement of nucleolar DSBs. Initial work from budding yeast has revealed that nucleolar DSB movements depend on the SMC5/6 complex, as well as its associated SUMOylation activities [19]. SMC5/6 is a ring-shaped protein complex with high structural similarity to the cohesin- and condensin- complexes and plays an important role in the maintenance of genome stability in different organisms (reviewed in [126]). Interestingly, recent work in human cells also revealed a role for the cohesin complex in the transcriptional silencing and movement of rDNA DSBs [111], suggesting that SMC complexes play a vital role in the movement and repair of rDNA DSBs in multiple organisms.

### 5.2 DSB movements and associated chromatin dynamics in heterochromatin

DSBs in the pericentromeric heterochromatic regions have also been observed to undergo specific dynamics and move outside of the heterochromatin domain in both Drosophila and mouse cells [20,67,80] (Figs. 3, 4B). Strikingly, live imaging in the presence of heterochromatic DSBs induced by X-ray irradiation also revealed movements to the nuclear periphery for a subset of these heterochromatic DSBs [75]. Additional imaging studies uncovered that these DSBs exert directed movement along the nuclear actin fiber to the nuclear periphery to continue their repair by HR [77,127]. Interestingly, these findings reveal striking analogies with DSB movements outside of nucleoli [19], which also depend on actin activities in concert with the nuclear- envelope associated LINC complex [111].

These outward DSB movements are thought to be necessary to preclude aberrant recombination between the many homologous repetitive DNA sequences present within one heterochromatin domain, and thereby prevent the accumulation of structural aberrations in chromosomes [20,75,101]. In line with this, inhibiting the movement of DSBs in constitutive heterochromatin in Drosophila leads to an increase in aberrant recombination, suggesting that these movements indeed prevent HR between the abundant homologous repeats present within this compact domain [20,75].

In Drosophila, live imaging of fluorescently-tagged HP1a revealed that heterochromatic DSB movements coincide with specific heterochromatin changes. In these experiments the induction of DSBs by X-ray IR resulted in the transient decompaction of the HP1a domain [20]. This decompaction could be due to the local removal of the canonical heterochromatin histone marks H3K9me2/3 at DSB sites by the histone demethylase dKDM4A, which was found to specifically promote DSB movement [16,73]. However, immunofluorescence imaging of mammalian cells following UV-induced photolesions or -DSB induction has revealed the maintenance, or even increases in H3K9me3 levels at constitutive heterochromatic break sites, while simultaneously observing local DNA decompaction [67,78,81]. These seemingly contradictory results indicate that there is likely a fine balance between H3K9me3 demethylation- and methylation- events as well as DNA decompaction, potentially dependent on the type of break, the repair pathway used, and the specific stage in the repair process.

In addition to heterochromatin decompaction, several chromatin proteins have been identified to play a role in the specific DSB dynamics in constitutive heterochromatin. For example, live imaging analysis of the Drosophila SMC5/6 complex has revealed its localization to constitutive heterochromatin before DSB movement [20]. Loss of SMC5/6 leads to defects in DSB relocalization outside the heterochromatin domain, indicating that this protein complex helps reorganize Drosophila heterochromatin before DSB movement [20,75,76]. Similar to the cohesin- and condensin- complexes, the SMC5/6 complex has recently been identified to exert DNA loop extrusion activities in vitro [128]. Since these SMC complexes have been described to play a role in DSB movement in nucleoli [19,111] as well as heterochromatin [20,75, 76], it is tempting to speculate that SMC loop-extrusion activities are required to overcome repetitive DNA- specific barriers to repairing DSBs.

Finally, earlier work in mouse cells has indicated a specific role for the ATM-dependent phosphorylation of Kap1 as well as local removal of the HP1β protein in heterochromatic DSB repair [70,72,74]. All these findings hint towards specific heterochromatin reorganization steps following DSB induction (reviewed in [54]).

### 5.3 DSB movements at centromeres

Studies focusing on DSB repair in both nucleoli and constitutive heterochromatin of mammalian cells suggest that the chromatin reorganization and DSB movements are likely specific for repair in the S and G2 phases of the cell cycle [67,111]. Interestingly though, mouse centromeres were found to promote DSB movement irrespective of the cell cycle phase, since CRISPR-Cas9 - induced DSBs move to the periphery of centromeres in both the G1 and G2 phase of the cell cycle [63,67] (Fig. 3). Which processes exactly promote the peripheral localization of centromeric DSBs remains currently unknown. However, a recent study did identify a role for the canonical centromere proteins CENP-A and HJURP, as well as transcription - dependent DNA-RNA hybrid formation in HR repair of DSBs at centromeres [63], indicating that centromeric DSBs depend on specific centromeric chromatin processes to enable their repair and maintain their stability.

In summary, what has become apparent from studies in a variety of different model systems is that several chromatin domains promote specific DSB - movements. These domain- specific DSB dynamics could be required to overcome certain physical restrictions imposed by chromatin domains and provide accessibility to DNA repair proteins. In addition to their specific biophysical properties [25,41,42], chromatin regions such as nucleoli and (peri) centromeres likely require more extensive DSB processing to overcome the presence of secondary structures associated with repetitive DNA [129–133]. The proper navigation of repair and specific processing steps may necessitate the movement of DSBs from their original locations. Indeed, it has been suggested that solely persistent, HR-dependent nucleolar DSBs require extensive rearrangements within the nucleoli [123]. In line with this, the peripheral movement of DSBs in yeast is exclusive to irreparable DSBs [134]. Together, this suggests that the degree of difficulty in repairing a DSB is directly proportional to the likelihood of undergoing specific movements and chromatin reorganization.

## 6 The impact of chromatin on DSB repair-pathway choice

Another major insight obtained from a variety of imaging studies is that different chromatin contexts promote differential DSB repair pathway choice. A major canonical controller of DSB repair pathway choice is the cell cycle [6]. Passaging from one cell cycle phase to the other is driven by changes in cyclin-CDK activity, which directly influence DSB repair-pathway choice [135–138]. In addition to cyclin-CDK driven changes, recent work has revealed that dilution of specific histone marks due to DNA replication can also directly guide DSB repair-pathway choice, where newly replicated chromatin serves as a recruitment platform specifically for HR proteins [139,140].

### 6.1 HR repair in G1 at centromeric- and nucleolar- DSBs

However, despite this canonical cell cycle-dependent regulation, several layers of evidence indicate that different chromatin regions can also directly influence DSB repair-pathway choice. For example, imaging of HR-protein recruitment revealed their presence at centromeric- and nucleolar- DSBs in both the G1 and G2 phase of the cell cycle [61, 63] (Fig.3). Moreover, the use of a new FISH technique that allows the visualization of both SSBs and DSBs at specific repetitive DNA sequences uncovered high levels of endogenous DNA damage at centromeres in non-dividing, quiescent cells. This endogenous damage was exacerbated upon loss of the HR protein RAD51 [66], suggesting that endogenously-induced centromeric DSBs depend on HR for their repair in G1 or G0. Interestingly, loss of RAD51 results in the increased usage of alternative DSB-repair pathways at centromeres in both dividing and quiescent cells, resulting in centromeric rearrangements [63,66]. Altogether, this work suggests that HR is a safer choice for repair at centromeres and prevents the use of alternative, more error-prone repair pathways that could be detrimental to the maintenance of centromeric structure and function (Fig.3).

### 6.2 DSB repair-pathway regulation in constitutive heterochromatin

In contrast to centromeres and nucleoli, DSBs in pericentromeric heterochromatin seem to follow the more canonical regulation of repair - pathway choice, where DSBs get repaired by NHEJ in G1 and by HR as well as NHEJ in G2 [67,69].

However, repair by HR in pericentromeric heterochromatin may require more extensive signaling to ensure its fidelity. Indeed, inhibition of the DNA-damage checkpoint kinase ATM in mammalian cells results in the specific accumulation of unrepaired heterochromatic DSBs in G2, indicating that heterochromatin might depend more heavily on ATM signaling to overcome the barrier for proper HR repair [9,70]. Moreover, work from both Drosophila- and mouse- cells has revealed that HR-repair proteins employ distinct spatiotemporal dynamics in constitutive heterochromatin domains to accommodate the recombination process. Live imaging analysis of a variety of HR proteins revealed that proteins important for the early stages of HR (e.g. DSB detection, end-resection) localize to DSBs within the heterochromatin domain, while the late-HR protein RAD51, important for homology search and the final steps of recombination, only localizes to DSB sites once they have been moved outside the heterochromatin domain [20, 67,75] (Fig.3).

The regulation of repair-pathway choice at heterochromatic DSBs in Drosophila depends specifically on the H3K9me2/3-demethylase dKDM4A [16,73]. Loss of dKDM4A results in an accumulation of HR proteins within the heterochromatin domain, suggesting that turn-over of heterochromatic histone marks is necessary for the regulation of co-ordinated HR-protein recruitment in heterochromatin [16]. Therefore, although DSB repair-pathway choice appears relatively conventional within constitutive heterochromatin, DSBs in this domain do undergo unconventional -movements and -HR protein recruitment and require specific chromatin changes to coordinate these processes.

The particular organization of DSB movements and late HR repair solely at the outer edges of heterochromatin may be specific to heterochromatin regions that contain numerous closely grouped repeats, such as in mouse- and Drosophila- cells. Unlike mouse- or Drosophila- cells, several human cell lines grown in a laboratory setting do not exhibit prominent chromocenters consisting of numerous repetitive sequences [44,101,141–143]. Indeed, in this heterochromatin arrangement with fewer clustered repeats, peripheral DSB movements are absent, and both early- and late- HR proteins are capable of localizing to DSBs within the heterochromatin domains [101]. This suggests that DSB movements and specific HR regulation are not inherently linked to repetitive heterochromatic sequences per se but likely depend on the specific molecular characteristics of heterochromatin, such as the level of clustering or the properties of heterochromatin proteins [101]. Restricting HR within regions with numerous clustered repeats would be logical since it would in theory be relatively easy for aberrant recombination to occur with many nearby homologous sequences. When there are fewer clustered repeats, it may not be necessary for such a movement mechanism to have evolved.

### 6.3 The nuclear periphery influences DSB repair pathway choice

Specific locations within the three-dimensional nuclear space can also contribute differentially to DSB repair pathway choice. For example, inducing a DSB at the, mostly heterochromatic, nuclear lamina in human cells prevents HR-protein recruitment and instead results in the use of the alternative end-joining (a-EJ) pathway [58](Fig.3). Indeed, DNA-sequencing of repair products following CRISPR-Cas9 - induced single DSBs at lamina-associated heterochromatin regions in human cells also revealed an increase in the use of a-EJ (i.e. MMEJ) when compared to canonical NHEJ [100]. These results suggest that DSBs in lamina-associated domains could depend more heavily on alternative repair pathways.

Interestingly though, not all locations at the nuclear periphery contribute to an increase in alternative repair-pathway usage. Several investigations conducted across various model systems have demonstrated that persistent double-strand breaks (DSBs) and stalled replication forks actually concentrate at the nuclear periphery for their safe repair. In such cases, the nuclear periphery establishes a conducive environment that facilitates the proper resolution of damaged sites (reviewed in [144]). Why the nuclear periphery is a potentially erroneous repair site for some DSBs but promotes HR for other DSBs likely depends on the specific site at the periphery. Indeed, localization of persistent DSBs to nuclear pore complexes in various model systems have been found to specifically promote HR repair [58,75,145,146], suggesting that the nuclear pore environment is important for safe repair, whereas other parts of the nuclear periphery may be more predisposed to undergo erroneous repair (Fig.3).

## 7 Summary and future outlook

The diverse chromatin environments in the eukaryotic nucleus, and the variability in their DNA sequence content, has led to the evolution of distinct repair mechanisms with differential dependencies on specific chromatin dynamics at the site of damage. This review highlights the imaging tools that have been employed to study repair in different chromatin contexts and has focused on the major findings that originated from this work. The use of inducible, locus-specific DNA damage tools in combination with fluorescent imaging of repair protein recruitment in different chromatin domains has led to breakthroughs in our understanding of the differential spatiotemporal dynamics of nucleolar-, heterochromatic- and centromeric- break sites.

However, the nucleus contains many more chromatin regions of which the repair response has not been studied in detail using high-resolution live imaging. Employing fluorescently-tagged chromatin proteins that specifically localize to certain chromatin regions, such as for example actively transcribed regions or polycomb bodies, could give more insights into the similarities and differences in repair between all the different chromatin regions. In addition, combining fluorescent markers for different chromatin domains within the same experimental set-up could also provide in-depth characterizations of the exact movements of DSBs; e.g. do heterochromatic DSBs always move to certain chromatin domains or do they accumulate randomly at the heterochromatin periphery? Moreover, different organisms, cell types and tissues have differential organizations of chromatin domains. Heterochromatin domains for example are assembled into different structures in different human cell-types as well as organisms [44], which raises the intriguing question whether there is a differential dependency of certain cell types or tissues on heterochromatic DSB movements and the maintenance of heterochromatin integrity [101]. It would be exciting to identify general principles, as well as differences, in the chromatin repair response in these varying contexts, and to understand what the evolutionary driving force is behind these differences.

Although previous work has identified many roles for specific chromatin changes at break sites [15,147], it remains difficult to determine the exact chromatin changes at specific genomic sites in space and time. For example, when does the peripheral movement and HR - protein recruitment of centromeric DSBs exactly start, and which chromatin changes accompany these movements? Similarly, actively-transcribed euchromatin regions depend heavily on HR for repair and are prone to actin-mediated DSB clustering in human cells [117,148,149]. However, the exact timing of HR-protein recruitment in specific cell-cycle stages and the concomitant chromatin- and DSB- dynamics at these actively transcribed regions within the three-dimensional nuclear space have not been elucidated in detail. Developing improved methods to visualize chromatin changes and DSB dynamics at high time- and spatial- resolution could enhance our understanding of the required chromatin changes for repair in different chromatin regions.

Recent work in cells employed photoactivated histones to track chromatin movements at break sites using structured-illumination microscopy in human [150]. This high-resolution imaging revealed reduced motions and chromatin condensation in regions distal to the damaged site, while revealing more dynamic chromatin closer to the break site. Combining this type of live image analyses with fluorescent markers for chromatin proteins or histone modifications (e.g. mint-bodies [151,152]) could in the future uncover the exact order of events of different chromatin changes at the damaged site, and their potential requirements in movement and repair.

Moreover, employing inducible DSB systems with real-time tracking of the turnover of newly synthesized- versus old- (parental) histones at DSBs, similar to what has been performed at UV-induced lesions in eu- and heterochromatin [81,153], could reveal new insights into the exact timing of histone turnover, as well as heterochromatic re-organization steps at DSBs. Combining these live-imaging methods with single-DSB induction [58,69] would be ideal, since that circumvents the problem of inducing many DSBs simultaneously, which could potentially impact outcomes due to for example limited amounts of repair proteins.

Another exciting development is the use of in vitro optical tweezers in combination with single-molecule live imaging [154] to assess repair-protein recruitment to ssDNA sites, thereby mimicking end-resected DSB sites [155]. These single-molecule analyses of repair-protein binding to damaged DNA structures may offer an intriguing new approach to assess how chromatin influences repair. By generating chromatinized arrays with different histone modifications [156–158] in the same optical tweezer set-up, one could address in an in vitro format the impact of each histone modification or chromatin protein on repair-protein binding to end-resected DSBs. An additional feature that has been introduced in similar optical-tweezer setups is the inclusion of specific secondary DNA structures [159]. By generating specific secondary structures that mimic those present in repetitive DNA sequences or at transcriptionally active regions, it could be possible to identify the exact influence of these secondary DNA structures on repair protein recruitment.

In short, live imaging studies of the repair process within different chromatin regions has resulted in exciting discoveries that have helped elucidate the specific nuclear properties that direct DNA repair. Future sophisticated in vitro and in vivo research incorporating high-resolution imaging will undoubtedly lead to new insights into our understanding of the impact of pre-existing chromatin domains on DNA repair.

Furthermore, the integration of insights obtained from live-imaging investigations with advancements in high-throughput sequencing methods, which facilitate comprehensive mapping of genetic -alterations and -modifications across the entire genome [160,161], is poised to significantly enhance our comprehension of how genome preservation mechanisms operate within diverse chromatin environments. This improved understanding will also shed light on the aberrations in these mechanisms observed in various diseases, opening up new prospects for targeted therapies in the future.

## 8 Material & methods

### 8.1 Immunofluorescence imaging of human cells

Retinal Pigment Epithelial (RPE-1) were maintained in Dulbecco’s Modified Eagle Medium (DMEM)/F-12 (Sigma-Aldrich, cat# D8062) supplemented with 10 % fetal bovine serum (FBS; Bodinco, cat# S007V20007), 1 mM alanyl-glutamine (Sigma-Aldrich, cat# G8541), and penicillin–streptomycin (Sigma-Aldrich, cat# P0781) in concentrations of 100 U/mL and 100 μg/mL, respectively. Irradiated cells were irradiated using the IBL437C (Schering CIS bio international, Cesium137 source) using a dosage time to reach 3 Gy. Cells were fixed with PEM-PFA buffer (1 % PFA, 10 mM EGTA, 1 M MgSO_4_, 100 mM pH 6.9 PIPES, 75 mM sucrose) for 4 min. Fixation was followed by 15 min of permeabilization with 0.5 % Triton X-100 and blocking for 1 h (3 % BSA, 0.2 % Tween-20). Cells were incubated with primary antibody (anti-HP1α (ab109028), anti-CREST (CS1058), anti-Nucleolin (ab190710) or anti-γH2AX (2577)) overnight at 4 °C. Cells were washed with block buffer before incubation for 1.5 h with secondary antibody (Invitrogen Alexa Fluor™ 488 Goat anti-Rabbit IgG A-11034, Invitrogen Alexa Fluor™ 568 anti-Human IgG A-21090, Invitrogen Alexa Fluor™ 647 Goat anit-Mouse IgG A-21236) at room temperature. Nuclei were stained with DAPI and coverslips were mounted to a glass slide using ProLong™ Diamond Antifade Mountant (ThermoFisher, P36962). Images were made with a DeltaVision RT Deconvolution Microscope (Applied Precision, LLC) and 60 × oil immersion objective. Images were deconvolved using softWoRx (LLC) and analyzed using Fiji (v2.9).

### 8.2 Live imaging of Drosophila melanogaster cells

Kc cells (embryonal *Drosophila melanogaster*) were maintained in CCM3 insect culture medium (HyClone; HYCLSH30065.02) supplemented with 1x Antibiotic Antimycotic Solution (Sigma-Aldrich; A5955). The Kc cells were cultured at 27 °C in a steam-saturated incubator. 3 days before imaging cells were transiently transfected with YFP-ATRIP and Cerulean-HP1a using TransIT-2020 (Mirus; MIR5404) following the manufacturer’s provided protocol, using a transfection agent to total DNA ratio of 2:1,2. The cells were plated in a µ-Slide 8 Well (Ibidi; #80821) before irradiation and subsequent imaging. Cells were irradiated with 5 Gy γ-irradiation using the IBL437C before live-imaging was started. Images were made with a DeltaVision RT Deconvolution Microscope (Applied Precision, LLC) and 60 × oil immersion objective with a time interval of 10 min. Images were deconvolved using soft-WoRx (LLC) and analyzed using Fiji (v2.9).

## Data Availability

Data will be made available on request.
